# Complicated atopic dermatitis in a healthy infant: Homemade walnut cream

**DOI:** 10.1002/ccr3.8198

**Published:** 2023-12-21

**Authors:** Bahareh Abtahi‐Naeini, Monir Sadat Emadoleslami, Mahsa Pourmahdi‐Boroujeni, Farnaz Bahrami‐Samani

**Affiliations:** ^1^ Pediatric Dermatology Division of Department of Pediatrics Imam Hossein Children's Hospital, Isfahan University of Medical Sciences Isfahan Iran; ^2^ Skin Diseases and Leishmaniasis Research Center Isfahan University of Medical Sciences Isfahan Iran; ^3^ Child Growth and Development Research Center Isfahan University of Medical Sciences Isfahan Iran; ^4^ Department of Pediatrics Children Imam‐Hossein Hospital, Isfahan University of Medical Sciences Isfahan Iran; ^5^ Student Research Committee Isfahan University of Medical Sciences Isfahan Iran

**Keywords:** atopic dermatitis, complementary and alternative medicine, eczema, topical treatment, walnut

## Abstract

**Key Clinical Message:**

Healthcare providers should educate patients on the appropriate use of topical agents and the potential risks associated with non‐standardized formulations, especially for infants and young children.

**Abstract:**

Complementary and alternative medicine (CAM) is an unconventional treatment method used alongside or in addition to conventional medical treatment methods to improve the healing process. Inappropriate administration of CAM can worsen the condition of diseases and have potential hazards for patients. Herbal therapy is one of the most famous and widely used CAMs in treating various skin disorders. In this case, we report a 4‐month‐old girl with atopic dermatitis who demonstrates ulceronecrotic lesions on her face and extremities besides sepsis 3 days after a walnut's homemade cream consumption. She was treated with intravenous clindamycin and wet‐to‐dry dressing to remove the scabs. This case report shows the potentially hazardous effects of misused traditional and homemade herbal therapy. It highlighted the need to pay particular attention when CAMs are used, especially for infants and young children.

## 1 INTRODUCTION

Atopic dermatitis (AD) is a common condition affecting 20% of the population during their lifetime. The treatment approach should alleviate and enhance symptoms through lifestyle modifications and medications. These include regularly applying moisturizers, topical corticosteroids, and calcineurin inhibitors, taking antihistamines, trying wet wrap therapy, avoiding triggers, and considering phototherapy.^1^, ^2^

Complementary and alternative medicine (CAM) refers to treatments not considered part of conventional medicine.^3^ Herbal remedies are commonly used as a complementary treatment for various diseases, such as cancer, rheumatic diseases, acquired immune deficiency syndrome (AIDS), and dermatological conditions such as psoriasis, acne, warts, and AD.^4^, ^5^ Usually, these treatments are cost‐effective and easily accessible. Consequently, many preferred herbal remedies over costly and possibly hazardous modern medications and techniques.^6^

Misused alternative medicine in treating these conditions can cause several problems, such as the recurrence of malignancies, diffuse dermatitis, localized or systemic contact dermatitis, and blisters on the skin.^3^, ^4^ Therefore, it is crucial to understand that the use of CAM should be approached with caution and only after consultation with a qualified healthcare provider. Herein, we present a case of a four‐month‐old girl who developed a hyperacute ulceronecrotic lesion on her face associated with sepsis formation after using a combination of homemade walnut and oil to treat AD.

## 2 CASE HISTORY

A four‐month‐old girl with atopic dermatitis presented to the emergency department (ED) with a 2‐day fever, irritability, and facial ulceronecrotic lesions over a previous erythematous patch. The infant had been scratching her face constantly for the past 2 months, resulting in impaired nighttime sleep quality and difficulty falling asleep. The infant was born full‐term via vaginal delivery and received the appropriate immunizations. She did not have any underlying medical conditions or any medication till admission. Neither the child nor her family had any recent travel or contact with contagious diseases.

**FIGURE 1:**
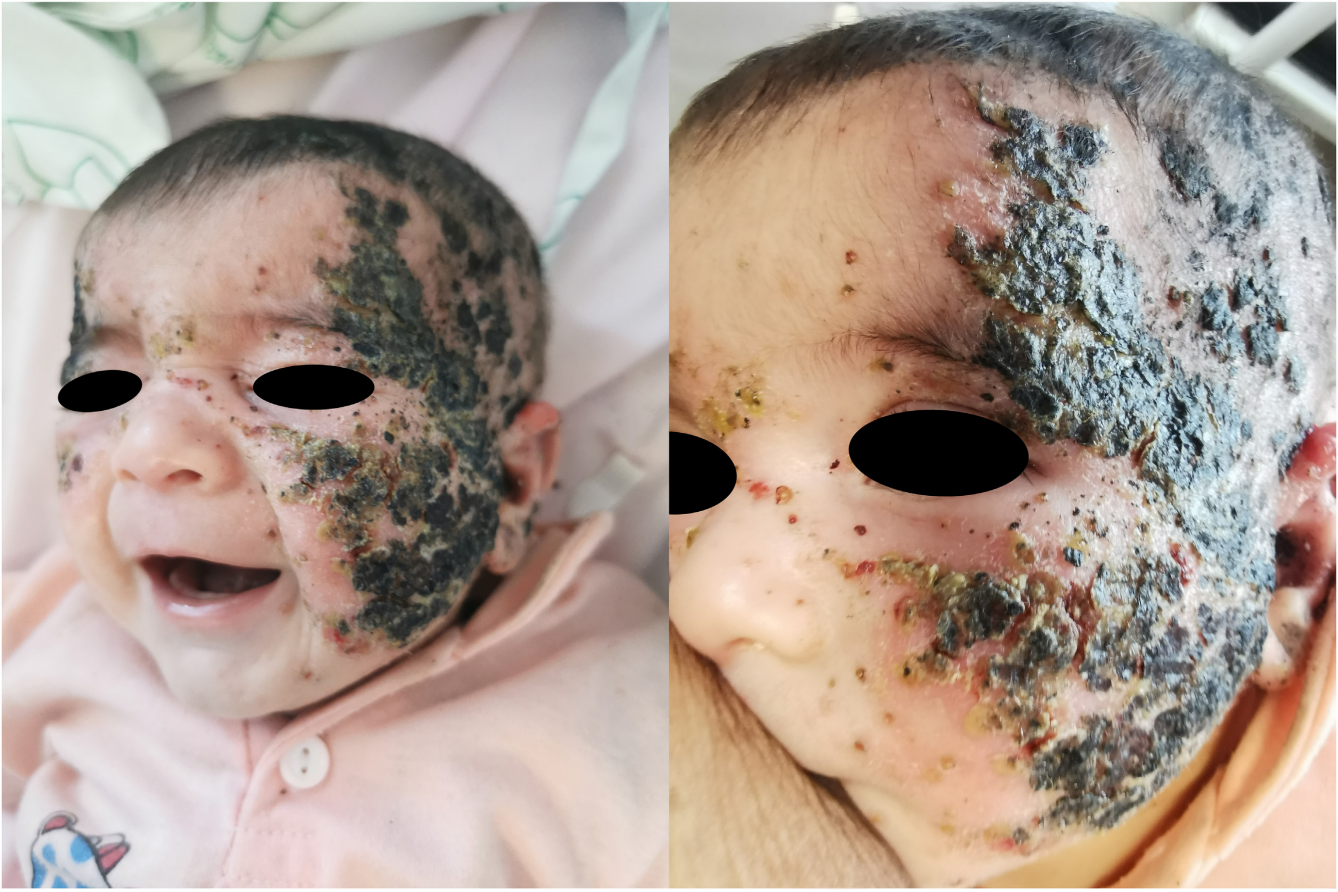
A 4‐month‐old girl with extensive facial ulceronecrotic lesions over erythematous patches of atopic dermatitis.

The mother applied a thick layer of homemade cream to the affected area of her face and limbs, which was formulated by a traditional experimental pharmacist and said to be a pure mix of crushed fresh walnut and olive oil. The cream was dominantly used on the face. It was the first time that walnut was used alongside olive oil on the skin, whereas in the past, solely oil was used with no complications. The ulceronecrotic lesions initiated from both cheeks expanded to the remainder of the face 3 days after using hand‐made cream, as shown in Figure 1.

**FIGURE 2:**
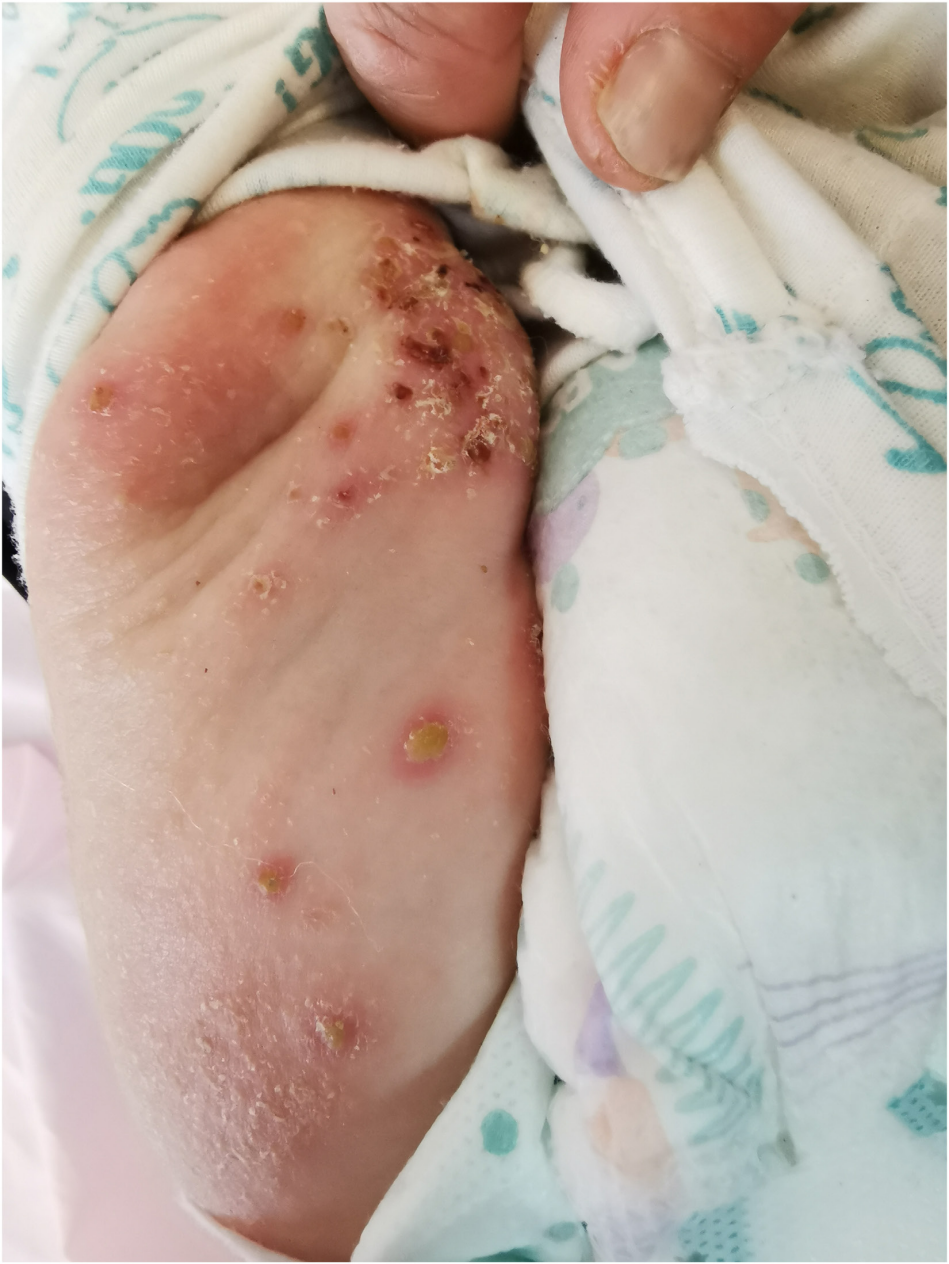
Diffuse pustular and crusted lesions all over the limb. AD, atopic dermatitis; CAM, complementary and alternative medicine; ED, emergency department; AIDS, acquired immune deficiency syndrome.

Physical examination on the day of admission demonstrated fever (up to 38.6°C and responsive to ibuprofen), respiratory rate of 40/min, a pulse rate of 130/min, irritability, and bilateral cervical lymphadenopathy. In addition to the facial involvement, there were scattered similar lesions on the trunk and extremities, as shown in Figure 2.

Workup was notable for a shift to left leukocytosis, elevated erythrocyte sedimentation rate, and C‐reactive protein. Procalcitonin was not checked. Blood culture was positive for Staphylococcus aureus. The patient was treated with intravenous clindamycin 10 mg/kg. The wet‐to‐dry dressing was used to remove the scabs.

After a few days, the ulceronecrotic lesions and infection exhibited regression without further complications. She was discharged with a prescription of a potent topical corticosteroid for a week, twice‐daily topical pimecrolimus (Elidel, SDZ ASM 981; Novartis Pharma AG) for a month, and 0.1% triamcinolone acetonide cream (Iran Darou Pharmaceutical Co.). In the 6‐month follow‐up, there was no evidence of infection recurrence.

## 3 DISCUSSION

We described an infant who developed hyperacute necrotic lesions on her face and sepsis after using homemade walnut and olive oil formulated as a cream for AD. Various studies have evaluated the efficacy of herbal remedies in treating AD, which were reported to have a wide range of responses and adverse effects. To our knowledge, such a catastrophic complication has not been reported.^3^, ^4^

Walnut has drawn broad interest in herbal therapies, considering it has various elements such as fibers, proteins, vitamins, and minerals. It is suggested to be helpful in viral, inflammatory, cardiovascular, and neurologic diseases due to its high quantity of omega‐3 fatty acids, polyphenols, antioxidants, and anti‐inflammatory and antiseptic properties. The walnut husks have an active ingredient called juglone, which improves fever, rheumatic pain, joint swelling, digestive problems, and skin disorders. It might also benefit patients with atopic dermatitis, either orally or as a topical medication, to reduce inflammation and improve skin hydration.^7^, ^8^

Along with this growth in the use of walnut as CAM, however, there is concern over its adverse effects, such as allergic reactions, contact dermatitis, digestive issues, and interactions with medications, and few studies reported notable side effects after applying walnut products.^7^, ^8^, ^9^

Topical use of walnut leaves by a 45‐year‐old woman to alleviate the pain and inflammation of the knee resulted in contact dermatitis.^8^ In addition, Neri et al.^10^ reported a couple of 4‐ and 5‐year‐old boys who developed skin‐pigmented lesions and contact dermatitis on their buttocks after playing with fresh green walnut husks in the garden of their nursery school.

As mentioned, herbal remedies, such as henna, olive oil, garlic, and lemon juice, are a part of CAMs. CAMs contain an exhaustive list of various treatments, including acupuncture, homeopathy, diets, probiotics, essential fatty acids, autologous blood injections, bio‐resonance treatment, aromatherapy, massage therapy, hypnotherapy, tea preparations, naturopathy, homeopathy, Ayurveda, and probiotics.^3^, ^5^

However, it should not be overlooked that reckless use of CAMs can have harmful consequences, such as liver damage and poisoning the body with substances like arsenic and Mercury. Additionally, the skin can be adversely affected, exacerbating preexisting skin conditions, the development of dermatitis or sweet syndrome, and, in severe cases, complications such as Stevens‐Johnson syndrome.^5^

Consuming unprocessed herbal products can interfere with their safety and weaken the skin's barrier, making it more susceptible to sunlight‐induced damage. To ensure the efficiency and safety of herbal remedies, it is essential to comprehend the quality of the medication, correct preparation methods and usage, and construct standardized preparations that have been clinically tested.^11^ Moreover, in our case, the cream was said to be a pure mix of olive oil and walnut husk. However, we cannot confirm this completely; a traditional experimental pharmacist synthesized it without evidence‐based medical education. It might have an additional unknown ingredient that could penetrate vulnerable skin affected by AD and aggravate the situation.

Despite the lack of scientific evidence on the therapeutic efficacy of some herbal remedies, people still use these treatments without proper medical supervision and formulation. Studies show that many patients do not inform their physicians about their use of CAMs.^3^ Even people with a high level of education frequently use CAMs without seeking medical advice.^5^

Uninformed use of CAMs can lead to irreversible consequences, especially for infants and young children. Therefore, healthcare providers should educate patients before using CAM, especially any use of these remedies for infants and young children.^4^ Our case emphasizes the potential hazards of using homemade herbal and traditional experimental remedies and the need for medical supervision when using CAMs.

## AUTHOR CONTRIBUTIONS

**Bahareh Abtahi‐Naeini:** Conceptualization; data curation; writing – review and editing. **Monir Sadat Emadoleslami:** Writing – review and editing. **Mahsa Pourmahdi‐Boroujeni:** Writing – review and editing. **Farnaz Bahrami‐Samani:** Data curation; writing – review and editing.

## FUNDING INFORMATION

None of the authors have made any financial disclosures. The authors of this article received no financial support for their research, drafting, or publishing.

## CONFLICT OF INTEREST STATEMENT

The authors declare that there is no conflict of interests regarding the publication of this paper.

## ETHICS STATEMENT

The protocol for this study was approved by the Ethics Committee of Isfahan University of Medical Sciences, Isfahan, Iran (IR.ARI.MUI.REC.1402.143).

## CONSENT

The Ethics Committee of the Isfahan University of Medical Sciences in Isfahan, Iran, approved this report's ethical content. Participation in the study was contingent upon signing a written informed consent form signed by the patient.

## DECLARATION OF INDEPENDENCY TO GOVERNMENT

We declare that none of the authors are employed by a government agency that has a primary function other than research and/or education. None of the authors have an official representative on behalf of the government.

## Data Availability

The data that support the findings of this study are not publicly available due to containing information that could compromise the privacy of our research participant but are available as requested.
